# Immunohistochemical and bioinformatic analysis of pannexin-1 expression in cutaneous basal and cutaneous squamous cell carcinomas

**DOI:** 10.1515/med-2026-1530

**Published:** 2026-09-30

**Authors:** Fikri Erdemci, Ömer Acer, Özden Yülek, Fırat Aşır, Hayat Ayaz, Inga Adanır, Fatih Taş, Ebru Çelik

**Affiliations:** Department of Histology and Embryology, Faculty of Medicine, Siirt University, Siirt, Türkiye; Department of Medical Microbiology, Faculty of Medicine, Siirt University, Siirt, Türkiye; Department of Pathology, Çanakkale Onsekiz Mart University, Çanakkale, Türkiye; Department of Histology and Embryology, Faculty of Medicine, Dicle University, Diyarbakır, Türkiye; Department of Dermatology, Siirt Training and Research Hospital, Siirt, Türkiye; Department of Histology and Embryology, Faculty of Medicine, Bandırma Onyedi Eylul University, Balıkesir, Türkiye; Department of Pathology, Siirt Training and Research Hospital, Siirt, Türkiye

**Keywords:** pannexin-1, cutaneous squamous cell carcinoma, basal cell carcinoma, integrative bioinformatics, immunohistochemistry

## Abstract

**Objectives:**

In this two-center study, we aimed to evaluate PANX1 expression in cutaneous squamous cell carcinoma (SCC) and cutaneous basal cell carcinoma (BCC) and to investigate molecular pathways and biological differences associated with PANX1 expression in these tumor types.

**Methods:**

In this study, the biopsy samples obtained for pathological examination from patients diagnosed with SCC and BCC were analyzed. The histochemical and immunohistochemical characteristics of the samples were evaluated. Bioinformatics and molecular docking studies were conducted for further analysis.

**Results:**

Digital image analysis using immunohistochemical staining and H-score evaluation on paraffin-embedded tissues revealed that PANX1 expression was significantly higher in SCC cases compared to BCC (p<0.001). In SCC, prominent cytoplasmic and membranous staining was observed in invasive tumor foci, while weak and homogeneous staining was detected in BCC samples. Bioinformatic analyses revealed that PANX1 interacts with proteins such as CASP1, CASP3, and P2RX7. However, Venn diagram analysis showed that CASP3 is the only common gene interacting with PANX1 and associated with SCC. No overlap was detected with BCC. Furthermore, molecular docking analyses revealed that ATP binds to PANX1 with high affinity and that caspase-3 establishes a stable interaction near the DVVD cleavage site of PANX1.

**Conclusions:**

Our two-center study demonstrated that PANX1 expression is higher in SCC compared to BCC and may play a role in the invasive phenotype. Bioinformatic analyses support the idea that PANX1’s interaction with CASP3 through inflammatory and apoptotic pathways may contribute to the progression of SCC. However, more comprehensive studies are needed to determine the diagnostic and prognostic value of PANX1.

## Introduction

The skin, which protects the body from damage caused by external factors, is the largest and most complex organ [1]. One of the basic physiological functions of the skin is to form a strong barrier against various harmful stimuli such as physical and chemical trauma, infectious agents and non-ionizing radiation to which the body is exposed [2]. In addition, genetic and epigenetic changes in epidermal cells and this continuous environmental exposure can contribute to carcinogenesis. As a result, the frequency of skin cancers is increasing, and they are among the most common malignancies globally [3], 4].

Skin cancer is a multifactorial disease that can develop due to strong genetic predisposition as well as the influence of environmental factors. Ultraviolet (UV) radiation is the most important environmental risk factors. Exposure to certain chemicals, stress, and some drugs further increase this risk [5]. UV radiation plays a key role in the development of both melanoma and non-melanoma skin cancers (NMSC). UV radiation has a central role in the pathogenesis of cutaneous basal cell carcinoma (BCC) and cutaneous squamous cell carcinoma (SCC) in particular [6]. Although NMSCs have a low potential for metastasis, their high incidence means they impose a significant cost on the global health system. However, underreporting in cancer registry systems causes the incidence of BCC and SCC to be shown as lower than it actually is in epidemiological statistics [7], [8], [9].

BCC is the most common type of skin cancer. It originates from epidermal epithelial cells or hair follicle stem cells [10]. At the molecular level, BCC develops as a result of the interaction between inherited genetic predisposition and somatic mutations [11]. The relationship between UV-induced oxidative stress and disordered molecular pathways leads to a higher incidence in sun-exposed areas [12]. Many cases can be effectively managed with surgical interventions or targeted therapies. Nonetheless, significant challenges may arise in the management of locally advanced (laBCC) or metastatic (mBCC) forms [13].

The second most widespread type of skin cancer in the world is cutaneous squamous cell carcinoma (SCC). Although most cases diagnosed at an early stage are curable, it has been observed that the death rate linked with SCC can reach values comparable to those of melanoma. [14]. BCC and SCC differ significantly in terms of biological behaviour, invasive potential, and prognosis, despite certain clinical and pathological similarities [15]. This emphasizes the necessity to differentiate the two tumor types at the molecular level and to identify specific biomarkers for them.

Pannexins (Panx1, Panx2, and Panx3) are glycoproteins that form channels in the cell membrane, playing a vital role in cellular communication by enabling the passage of small signaling molecules [16], 17]. In particular, ATP release via PANX1 is effective in both physiological and pathological processes and is associated with mechanisms such as cellular proliferation, inflammation, and cell death [18]. It has been reported that pannexin channels not only exhibit channel-mediated functions but can also exert channel-independent effects through interactions with various signaling pathways [17]. Therefore, it is thought that changes in pannexin expression may be associated with tumour development and progression [19].

The literature indicates that proteins of the Pannexin family exhibit variable expression profiles in tumours of different organs and tissues; in some contexts, they may act as tumour suppressors, while in others, they may play a role in promoting tumour progression [20], 21]. On the other hand, there are extremely limited studies comparing Pannexin expression in non-melanoma skin cancer, especially BCC and SCC, and more investigation is required.

The purpose of this study was to compare the expression patterns of PANX1 protein in BCC and SCC using immunohistochemical methods, as well as to examine the characteristic histopathological features of both tumour types using hematoxylin-eosin (H&E) sections. The relationship between PANX1 expression levels and tumor cell differentiation, invasive capacity, and histopathological parameters was investigated. This aimed to provide evidence-based data on the role of PANX1 in the pathogenesis of non-melanoma skin cancers and its potential utility as a prognostic biomarker in clinical practice.

## Materials and methods

### Tissue preparation

This retrospectively designed study included biopsy samples taken for pathological examination from patients diagnosed with squamous cell carcinoma or basal cell carcinoma who presented to Siirt Training and Research Hospital and Çanakkale 18 March University Faculty of Medicine between 2020 and 2025. Ethical approval for this study was obtained from the Siirt University Ethics Committee for Non-Invasive Clinical Research (approval date: 03.12.2025; approval number: 2025/01/12/4), and the study was conducted in accordance with the Declaration of Helsinki.

All tissue samples were fixed in a 10 % neutral formalin solution. After fixation, the tissues were washed in tap water, then passed through a series of ethanol and xylene stages at increasing concentrations (50 %, 70%, 80%, 90%, 96 %, and absolute ethanol) and embedded in paraffin. Sections 5 µm thick were cut from the paraffin blocks and mounted on slides. The sections were prepared using a microtome (Leica RM2265, Wetzlar, Germany).

### Immunohistochemical staining

Sections were obtained from paraffin blocks of squamous cell carcinoma and basal cell carcinoma biopsies, deparaffinized in xylene for 2 × 15 min, then passed through a series of decreasing ethyl alcohols and placed in distilled water and washed with phosphate-buffered saline (PBS). For antigen retrieval, some sections were heated in EDTA buffer solution (pH: 8.0; catalogue no.: ab93680, Abcam, Cambridge, USA) by microwave method and cooled to room temperature before being stored in PBS. Endogenous peroxidase activity was blocked with 3 % hydrogen peroxide solution (catalog no: TA-015-HP, Thermo Fisher, USA) for 20 min. An ultra-V blocking solution (catalog no. TA-015-UB, Thermo Fisher, USA) was applied to the sections washed with PBS for 7 min to prevent non-specific binding. Then, the sections were incubated with the PANX1 antibody (catalog no. epr28631-135, Abcam, US) at +4 °C overnight. The next day, the sections were brought to room temperature and washed with PBS, then incubated with secondary antibody for 14 min. Then, streptavidin-peroxidase solution (catalog no. TS-015-HR, Thermo Fisher, USA) was applied and kept for 15 min. The immune reaction was developed with diaminobenzidine (DAB) substrate (catalog no: TA-001-HCX, Thermo Fisher, USA). Counterstaining was performed with Harris hematoxylin for 40 s, then the sections were washed in tap water for 5 min. Sections were passed through a series of increasing concentrations of ethyl alcohol, dried, covered with Entellan and coverslipped. All preparations were viewed with a Zeiss Imager A2 light microscope; digital analyses were performed using Zen 3.0 software (Germany).

### Protein–protein interaction and KEGG pathway enrichment analysis

Protein–protein interaction (PPI) analysis of PANX1 was conducted utilizing the STRING (Search Tool for the Retrieval of Interacting Genes/Proteins) database, which integrates established and anticipated protein–protein interactions from various sources, encompassing experimental data, computational prediction techniques, and public text repositories [22]. The organism was assigned the name *Homo sapiens*, and the interaction network was constructed utilizing a minimum necessary interaction score of 0.4 (medium confidence).

Genes linked to cutaneous squamous cell carcinoma and basal cell carcinoma were obtained from the GeneCards database, an extensive library of human genes and their functional annotations [23]. To enhance the specificity of gene sets linked to disease, genes with a relevance score greater than 10 were selected. To find overlapping genes between PANX1-interacting proteins and genes linked to disease, Venn diagram analysis was utilized.

A functional enrichment analysis of PANX1-related genes was carried out using the ShinyGO platform [24]. To evaluate pathway enrichment, we utilized the Kyoto Encyclopedia of Genes and Genomes (KEGG) database, which provides carefully chosen biological pathways linking genes to molecular activities and cellular processes [25]. Pathways were considered statistically significant if their false discovery rate (FDR) adjusted p-value was<0.05.

All bioinformatics results and visual representations depicted in Figure 4 were exclusively produced by the authors for this work and have not been replicated or altered from previously published figures.

### Molecular docking

#### Protein–protein docking analysis

In addition to ligand-protein interactions, protein-protein docking was performed to investigate the interaction between caspase-3 and PANX1. In this study, the crystal structure of the human caspase-3 enzyme was obtained from the Protein Data Bank (PDB) under the code 1NME [26]. The AlphaFold Protein Structure Database [27] was used to obtain the three-dimensional structure of the PANX1 protein. It was refined by removing unnecessary molecules using the chimera-1.19 program.

The produced structures were submitted using the integrated modelling platform HADDOCK v2.4 web server [28]. Interaction regions were defined according to literature data, and “active residues” were identified. The catalytic residues His121 and Cys163 [29] of caspase-3 and the PANX1 C-terminal cleavage site (residues 376–379; DVVD) were defined as active residues [30].

The HADDOCK scores were used to rank the docking solutions, and the complex with the highest score was chosen for further analysis (cluster-1). The amino acid residues involved in the interaction interface were found by visualizing this complex with UCSF Chimera [31].

#### Protein–ligand docking

This paper references the 3D structure of PANX1 (PDB ID: 7F8J) was obtained from the RCSB Protein Data Bank. Following the removal of heteroatoms and water molecules from the three-dimensional structure, polar hydrogen atoms were then incorporated. The PubChem database was used to get the molecular structure of adenosine triphosphate (ATP), which acts as a ligand. The structures were prepared before docking by eliminating heteroatoms and water molecules, then adding polar hydrogen atoms and assigning the proper charge. The AutoDock Vina algorithm [32] is used by the PyRx virtual screening tool [33] for docking simulations. A blind docking method was employed to identify putative binding sites on the protein surface. The optimal binding conformation was determined by the lowest binding energy. The data were subsequently visualized and analyzed via Discovery Studio Visualizer from Dassault Systèmes, and ligand–protein interactions were assessed by the “Analyze Ligand Interactions” module.

### Statistical analysis

The sample size and statistical power of the study were evaluated using the G*Power (v3.1.9.7) program. In the power analysis, independently of the prior literature data, a moderate effect size (h=0.40) was used according to Cohen’s statistical standards, and a clinically significant 20 % difference in ratio (Proportion 1=0.40, Proportion 2=0.60) was predicted between the two tumor groups [34]. In the *a priori* power analysis, based on a 5 % type I error margin (alpha=0.05), 80 % target test power, and an asymmetric distribution ratio between groups (allocation ratio=0.32), it was determined that, according to Fisher’s exact test model, the study should be completed with a total of 271 cases, consisting of at least 205 BCC and 66 SCC. The 231 BCC and 74 SCC cases used in our study (a total of 305 cases) meet this minimum sample size criterion. In the post-hoc power analysis performed based on the available case numbers, the statistical power achieved in the study was determined to be 84.37 % (Power=0.844) [35].

Statistical analyses were performed using SPSS software (version 25, IBM Corporation, Armonk, New York, USA). The Kolmogorov-Smirnov test was used to examine whether the continuous variables (Age, Tumor Size, and Invasive Depth) included in the study showed a normal distribution. The analysis revealed that none of the variables exhibited a normal distribution (p<0.05). Therefore, the Mann-Whitney U test, a non-parametric analysis method, was used to compare continuous variables between gender groups (female and male).

The Shapiro-Wilk test was used to assess the normality of the H-score distribution obtained using QuPath. Since the data obtained in our study did not show a normal distribution, a non-parametric analysis was performed. The Mann-Whitney U test was performed to evaluate the differences in PANX1 H-scores between BCC and SCC tumors. The results obtained were expressed as medians (interquartile range, IQR). p values ​​less than 0.05 were considered statistically significant.

## Results

### Demographic and clinical parameters

Demographic and clinical parameters are presented in Tables 1 and 2. The tables present parameters such as mean age, gender, tumor size, tumor location, and depth of tumor invasion in patients diagnosed with BCC and SCC.

**Table 1:** BCC demographic and clinical parameters.

		Male (n=120)	Female (n=111)	Total (n=231)	p-Value
Age	Mean	69.51 ± 13.40	68.56 ± 13.50	69.06 ± 13.43	p=0.521
	Min-max Ist.	36–96	36–99	36–99	
Tumour size, cm	Mean	0.98 ± 0.44	0.93 ± 0.34	0.96 ± 0.40	p=0.903
	Min-max Ist.	0.3–2.60	0.2–2.00	0.2–2.60	
Invasion depth, cm	Mean	0.28 ± 0.118	0.27 ± 0.13	0.277 ± 125	p=0.698
	Min-max Ist.	0.10–0.65	0.10–0.60	0.10–0.65	
Tumour location	Frontal region	8	3	11 (4.8 %)	
	Cervical region	4	2	6 (2.6 %)	
	Nasal region	37	46	83 (35.9 %)	
	Labial region	9	11	20 (8.7)	
	Eye region	5	10	15 (6.5 %)	
	Brow region	3	1	4 (1.7 %)	
	Ear region	6	4	10 (4.3 %)	
	Dorsal region	4	1	5 (2.2 %)	
	Scalp region	17	10	27 (11.7 %)	
	Cheek region	27	23	50 (21.6 %)	
	Total	120	111	231 (100 %)	

**Table 2:** SCC demographic and clinical parameters.

		Male (n=49)	Female (n=25)	Total (n=74)	p-Value
Age	Mean + SD	71.918 ± 11.41	72.68 ± 14.81	72.175 ± 12.56	p=0.510
	Min-max Ist.	50–93	41–92	41–93	
Tumour size, cm	Mean	1.42 ± 0.91	1.125 ± 0.58	1.32 ± 0.82	p=0.330
	Min-max Ist.	0.10–4.60	0.13–2.40	0.10–4.60	
Invasion depth, cm	Mean	0.38 ± 0.26	0.35 ± 0.20	0.36 ± 0.22	p=0.768
	Min-max Ist.	0.10–1.20	0.10–1.10	0.10–1.20	
Tumour location	Frontal region	2	0	2 (2.7 %)	
	Cervical region	3	0	3 (4.1 %)	
	Nasal region	7	5	12 (16.2 %)	
	Labial region	7	6	13 (17.6 %)	
	Hand region	4	5	9 (12.2 %)	
	Scalp region	11	2	13 (17.6 %)	
	Brow region	3	0	3 (4.1 %)	
	Ear region	7	0	7 (9.5 %)	
	Cheek region	1	3	4 (5.4 %)	
	Facial region	4	4	8 (10.8 %)	
	Total	49	25	74 (100.0 %)	

The Mann-Whitney U test was applied to compare age, tumor size, and invasion depth parameters between gender groups in BCC tumors. According to the analysis results, no statistically significant difference was found between genders in terms of age (p=0.521), tumor size (p=0.903), and Invasion depth (p=0.698) (Table 1).

The Mann-Whitney U test was applied to examine the distribution of age, tumor size, and invasion depth parameters among gender groups in SCC tumors. The analysis revealed no statistically significant differences between genders in terms of age (p=0.510), tumor size (p=0.330), and Invasion depth (p=0.768) (Table 2).

### Histopathological findings

Examinations of sections stained with hematoxylin-eosin revealed distinct morphological features consistent with BCC and SCC (Figure 1). In Figure 1A and B (BCC), basaloid tumor nests originating from and attached to the epidermis, extending towards the superficial dermis, were detected at low magnification. The detected tumor islands were found to be well-defined and embedded in a fibromyxoid stroma. The neoplastic cells, at higher magnification, were small and basophilic, with hyperchromatic nuclei and a small amount of cytoplasm. Additionally, a prominent peripheral palisade arrangement of tumor cells was detected. Furthermore, characteristic stromal retraction clefts were detected between the tumor islands and the surrounding connective tissue, supporting the diagnosis of BCC.

**Figure 1:**
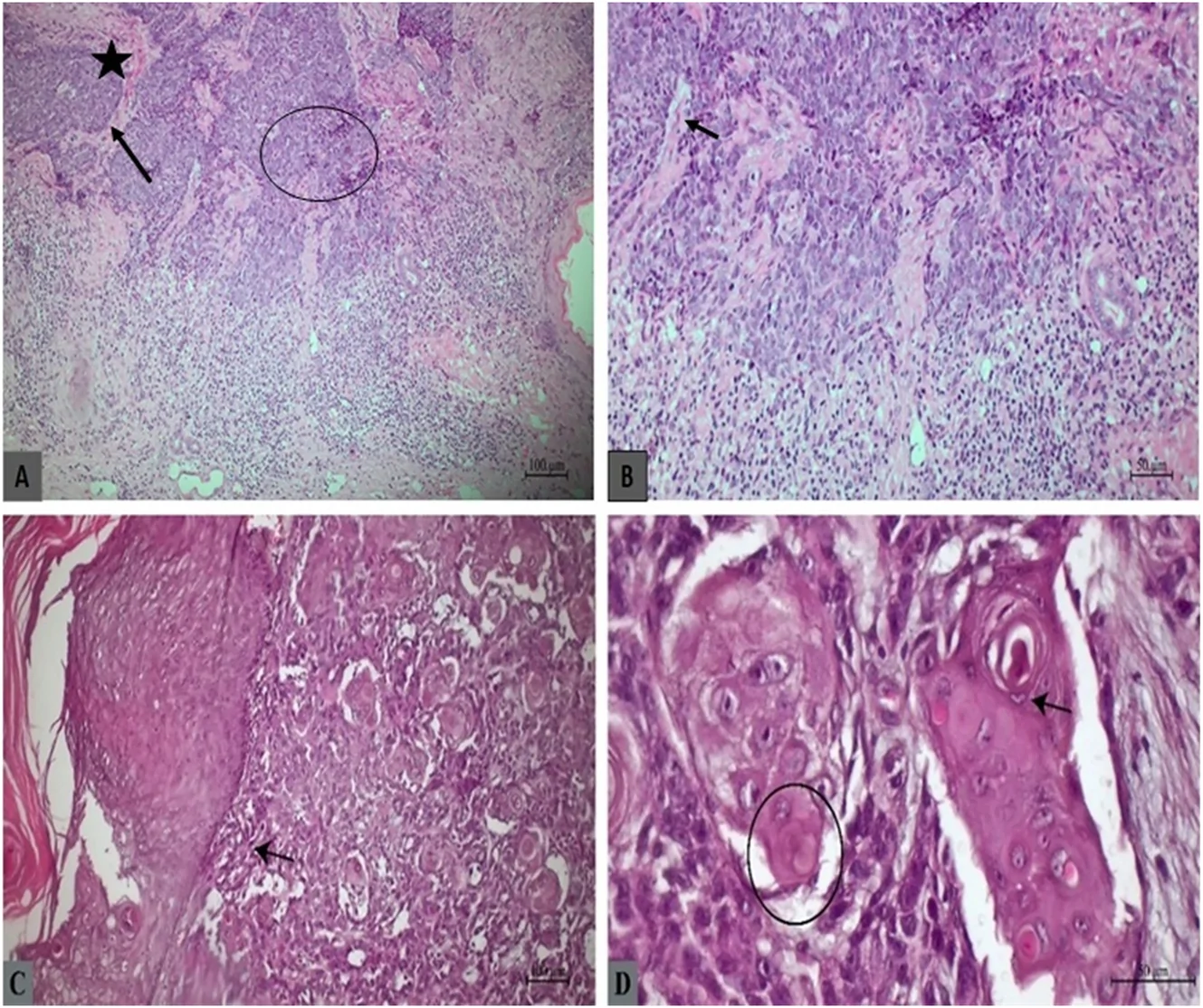
Histopathological features of BCC and SCC in hematoxylin and eosin (H & E)-stained sections. (A) Low magnification view of BCC; basaloid tumor nests extending from the epidermis into the dermis (circle), fibromyxoid stroma (asterisk), and peripheral palisading of basaloid cells (arrow). (B) High magnification view of BCC: stromal retraction clefts between tumor nests and the surrounding stroma (arrow). (C) Low magnification view of SCC: irregular invasive nests of atypical squamous cells extending into the dermis (arrow). (D) High magnification view of SCC; keratin pearl formation (circle) and atypical squamous cells with eosinophilic cytoplasm (arrow). Scale bars: 100 µm (A, C); 50 µm (B, D).

In Figure 1C and D (SCC), at low magnification, irregularly shaped invasive cords extending into the dermis and atypical squamous cell nests were detected. The overlying epidermis showed an irregular architecture with downward-extending proliferative extensions. At higher magnifications, the cytoplasm of the tumor cells was found to be eosinophilic, nuclear pleomorphism was prominent, and the presence of nucleoli was noticeable. The tumor foci showed keratinization and the formation of keratin pearls. In addition, intercellular bridges were detected in squamous cell differentiation areas. These findings are consistent with invasive squamous cell carcinoma.

### Pannexin-1 immune staining findings

Differential expression of PANX1 between BCC and SCC was identified using immunohistochemical investigation, as represented in Figure 2. In BCC analyses, PANX1 expression was frequently observed to be mild or moderate, primarily localized in basaloid tumour nests. It was observed that expression was primarily cytoplasmic with localized membranous highlighting. The peripheral stromal components showed negligible or negative expression. The overlying epidermis showed slight background positivity (Figure 2A and B).

**Figure 2:**
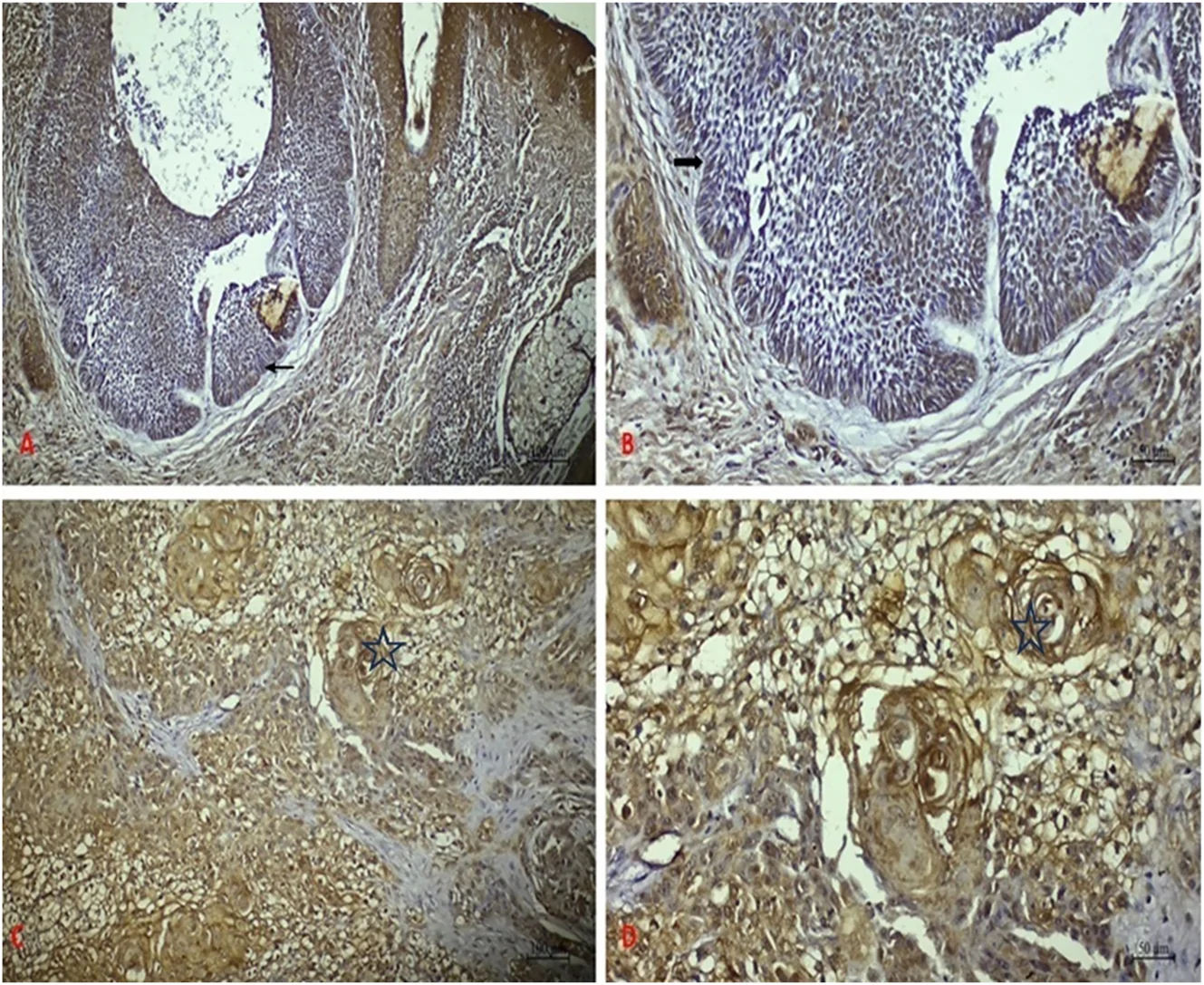
Immunohistochemical expression of PANX1 in BCC and SCC: (A, B) BCC sections showing generally weak to moderate PANX1 expression in basaloid tumor nests (arrow) and minimal stromal staining. (C, D) SCC sections showing stronger and more widespread cytoplasmic and membranous PANX1 expression in invasive squamous nests and especially around keratin pearls (asterisks). Scale bars: 100 µm (A, C); 50 µm (B, D).

It was observed that SCC samples had higher and more extensive PANX1 expression. Invasive squamous nests, squamous differentiation regions, and the vicinity of keratin pearls all showed strong cytoplasmic and membrane expression. The staining intensity and distribution were significantly higher in SCC compared to BCC. Furthermore, increased PANX1 expression was detected in squamous neoplastic cells (Figure 2C and D).

Semi-quantitative digital image analysis using QuPath revealed significantly higher PANX1 expression in SCC compared to BCC (Figure 3). The median H-score was found to be significantly elevated in SCC samples, with a wider distribution and higher upper quartile values. However, relatively low and homogeneous staining intensity was observed in BCC samples. Statistical evaluation showed a significant difference between the groups (Mann-Whitney U test, p<0.001).

**Figure 3:**
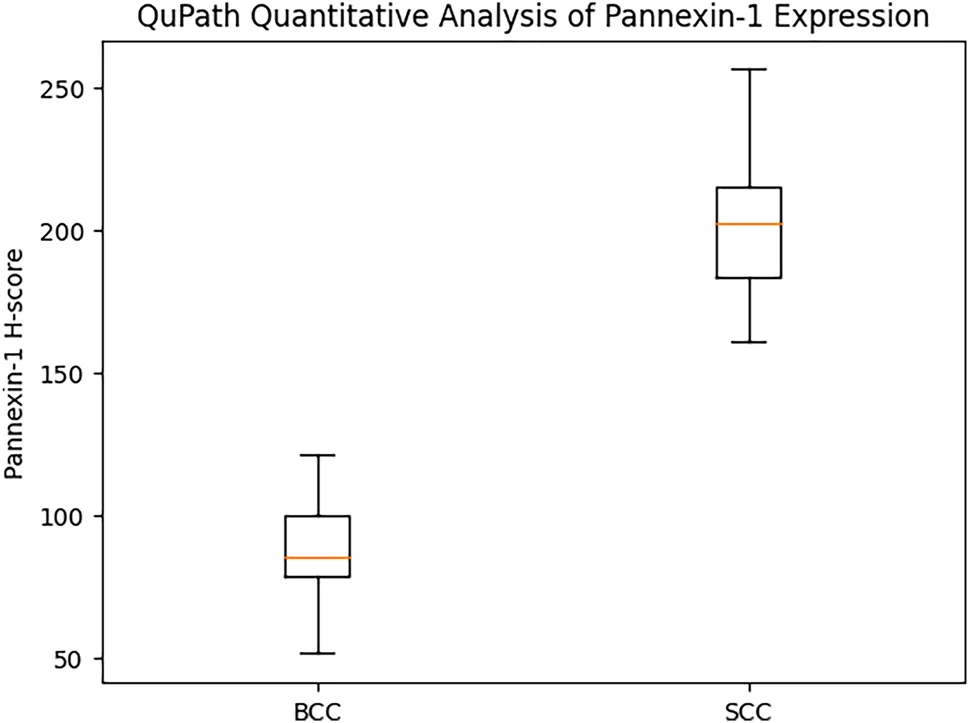
Quantitative comparison of PANX1 immunoreactivity between BCC and SCC; box plot showing H-scores obtained by QuPath digital image analysis: SCC exhibited significantly higher PANX1 expression than BCC (Mann-Whitney U test, p<0.001). Boxes represent interquartile range (IQR), the central line indicates the median, and whiskers represent minimum and maximum values.

### Functional enrichment analysis of PANX1-Associated genes

Potential molecular processes associated with PANX1 in cutaneous skin cancers were investigated using integrative bioinformatics analysis. The results of the analysis are shown in Figure 4A–C Various proteins with which PANX1 interacts were identified using protein-protein interaction analysis with the STRING database. These included proteins associated with purinergic signaling and inflammatory pathways such as P2RX7, CASP1, and CASP3 (Figure 4A).

**Figure 4:**
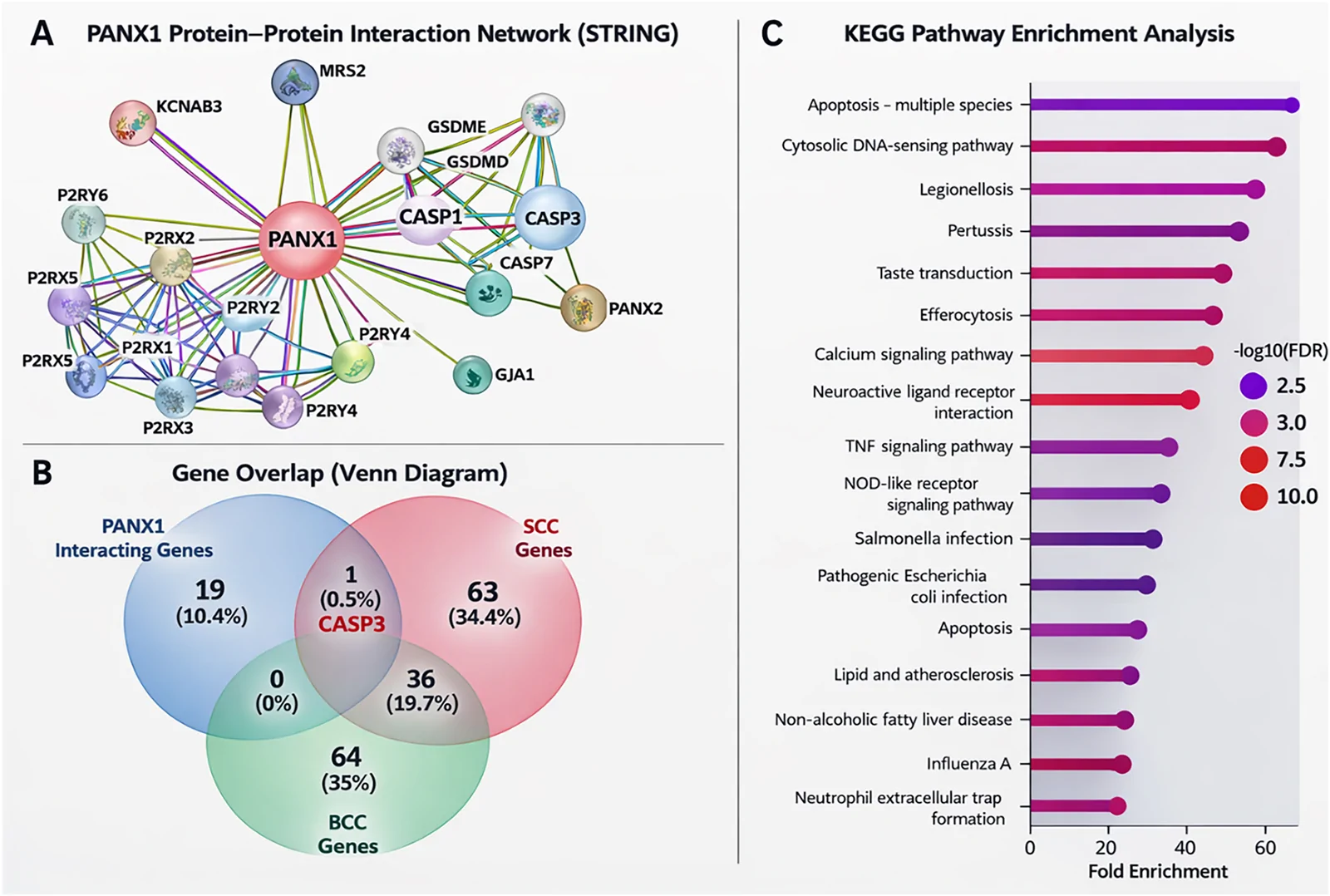
Bioinformatic analysis of PANX1-associated pathways in cutaneous cancers. (A) Proteins involved in purinergic signaling, apoptosis, and inflammatory processes are highlighted in the protein–protein interaction network of PANX1 and its interacting proteins that was created using the STRING database. (B) A Venn diagram illustrating the intersection of PANX1-interacting proteins and genes linked to SCC and BCC as sourced from the GeneCards database. (C) Apoptosis, TNF signaling, calcium signaling, and inflammatory pathways were significantly enriched in the KEGG pathway enrichment analysis of PANX1-associated genes carried out using ShinyGO.

The relationship between PANX1 and genes associated with skin cancer was analyzed using Venn diagrams. Gene sets associated with squamous cell carcinoma and basal cell carcinoma of the skin were retrieved from the GeneCards database (Figure 4B). The common gene at the intersection between genes associated with SCC and proteins interacting with PANX1 was identified as CASP3. This suggests that PANX1-mediated signaling and apoptotic pathways may be related in squamous cell carcinoma of the skin.

Functional enrichment analysis of PANX1-related genes was performed using ShinyGO with pathway descriptions obtained from the KEGG database. KEGG pathway analysis revealed substantial enrichment in multiple pathways associated with inflammation and programmed cell death, specifically apoptosis, TNF signaling, calcium signaling, and cytosolic DNA-sensing pathways (Figure 4C). The findings imply that PANX1-related signaling networks might be involved in regulating apoptosis and inflammatory responses in cutaneous skin cancers.

### Molecular docking results

#### Protein–protein docking

Protein–protein docking analysis suggested a stable interaction between PANX1 and Caspase-3 (Figure 5A). The most advantageous binding position and interaction energy were shown by the top-ranked docking cluster. Van der Waals interactions and electrostatic forces were found to be the interaction’s main driving forces, suggesting a strong and particular binding configuration.

**Figure 5:**
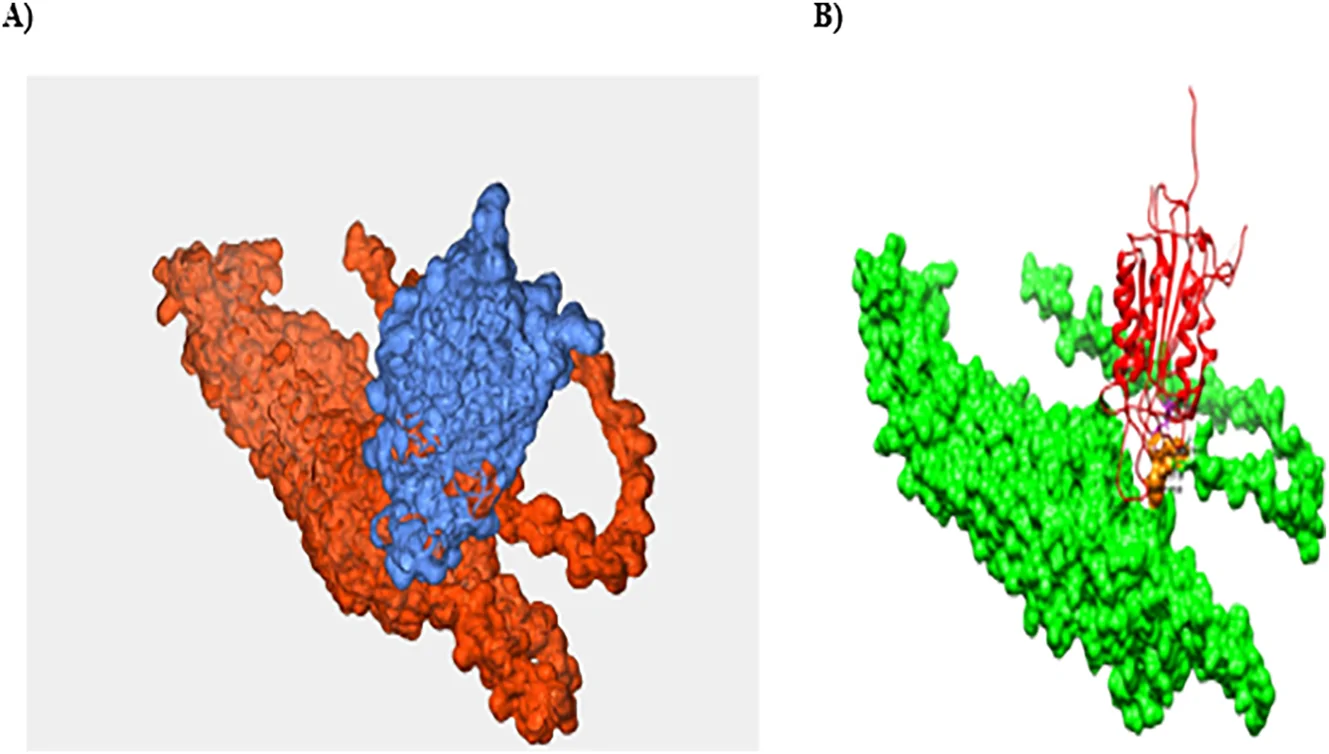
Analysis of protein-protein docking between PANX1 and Caspase-3. (A) Top-ranked docking model (Cluster 1) of the PANX1–Caspase-3 complex produced using HADDOCK v2.4. PANX1 is represented in red, while Caspase-3 is represented in blue. Structural image of the docked complex in UCSF Chimera 1.9, featuring PANX1 in green and Caspase-3 in red. Potential binding sites and interaction surfaces between the two proteins are represented by the orange and purple regions. Notably, the catalytic residues His121 and Cys163 of caspase-3 are situated near the C-terminal portion of PANX1, specifically the DVVD cleavage site.

Figure 5B depicts the Chimera-based structural visualization analysis. A specific binding interface has been established between Caspase-3 catalytic residues (His121 and Cys163) and the DVVD cutoff site in PANX1’s C-terminal region.

#### Protein–ligand docking

It was determined that the PANX1-associated protein network is significantly enriched in terms of ATP-sensitive processes and purinergic signaling pathways. Given these results, ATP has been utilized as a likely natural ligand in molecular docking studies designed to investigate its prospective interaction with PANX1. According to the molecular docking analysis, ATP had a binding affinity of −7.2 kcal/mol and fit properly into the PANX1 binding cavity. The ligand’s deep pocket location determined the stability of the binding conformation.

It was determined that the positively charged Lys18 and Arg128 residues have strong electrostatic interactions with the negatively charged phosphate groups of ATP. Hydrogen bond interactions with Glu19 and Asp14, thought to aid in ligand stability, were detected. Additionally, the aromatic residue Tyr121 participated in hydrophobic interactions, supporting ATP binding within the pocket (Figure 6).

**Figure 6:**
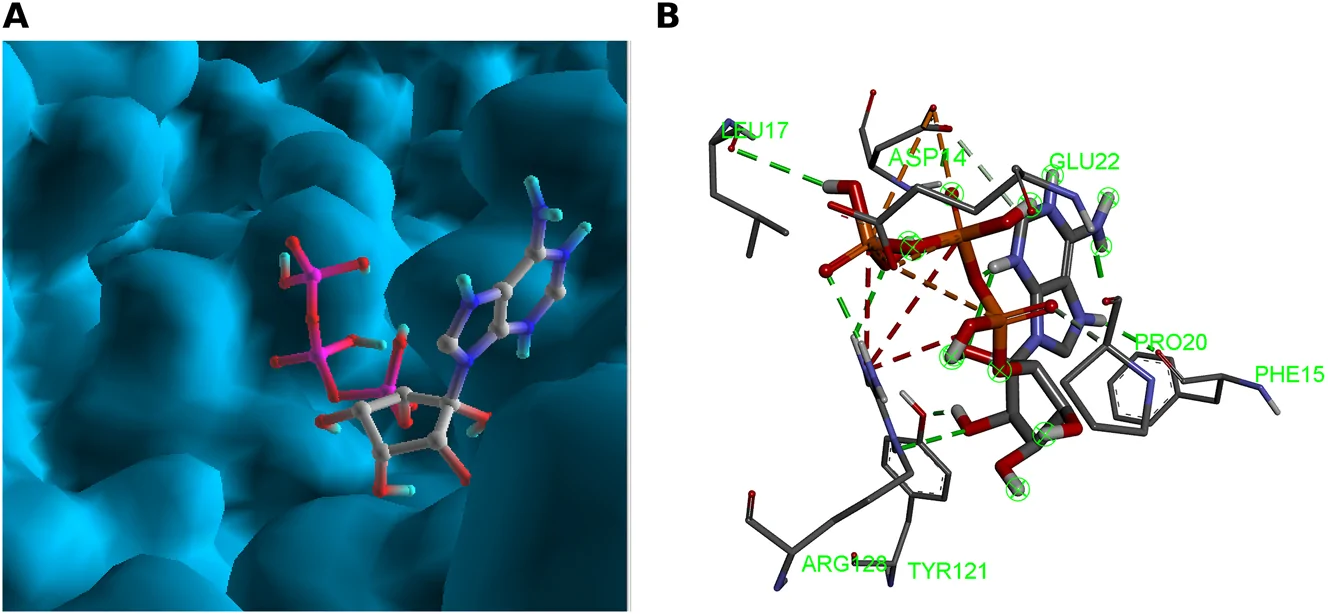
The binding of ATP to the PANX1 cavity’s surface is depicted in Figure 6. Important interactions involving the residues Lys18 and Arg128 (electrostatic), Glu19 and Asp14 (hydrogen bonding), and Tyr121 (hydrophobic) are indicated by colored lines. A brief summary of the many types of interactions is provided in the legend.

The functional involvement of PANX1 in purinergic signaling is consistent with the general binding mechanism, which suggests that ATP is stabilized by a mix of electrostatic, hydrogen bonding, and hydrophobic interactions.

## Discussion

The main finding of this study is that PANX1 expression is significantly higher in SCC compared to BCC. In this analysis, based on a large, multicenter patient series, PANX1 expression was evaluated using immunohistochemical and bioinformatic methods. In comparison to BCC cases, our results showed a markedly higher expression of PANX1 in SCC cases. The detected difference was statistically significant and consistent, according to quantitative results from the H-scoring. The results suggest that PANX1 expression may be a prospective molecular marker that highlights differences in biological behaviour among subtypes of keratinocyte tumours.

The reliability and generalizability of the findings are enhanced by the fact that the study was conducted at two distinct centers and involved a large patient group. Consistent PANX1 expression in the two centers suggests that the difference seen across tumour groups is not attributable to technical or sampling issues but may reflect a tumor-specific biological feature. This suggests that PANX1 could be a molecule involved in the biological distinction between BCC and SCC.

The tumour known as BCC is distinguished by its slow progression pattern and low potential for metastasis [36], 37]. Local tissue invasion is the hallmark of BCC, and reported incidence rates range from 0.0028 to 0.55 % [38]. However, BCC can result in ulceration, deformity, or recurrence if treatment is delayed or insufficient [39]. Basaloid cell nests and clusters are arranged in a peripheral palisade pattern in the histological appearance of BCC. These cells, which are characterized by hyperchromatic nuclei and minimal cytoplasm, are frequently associated with fibromyxoid stroma and stromal atrophy [40]. In BCC, nuclei that exhibit a palisade or fence-like configuration within the cellular layer surrounding the tumour mass are deemed pathognomonic [37].

Research has demonstrated the strong stroma dependence of BCC tumours. This implies that unless they carry some of their stroma, they do not metastasize to other tissue regions [41]. The low expression of PANX1 detected in BCC appears to be in agreement with this limited invasive behaviour. Considering that PANX1 channels regulate ATP release, paracrine signaling, tumour immunity, progression, and prognosis and contribute to the tumour immune microenvironment [42], 43], the low levels of PANX1 expression found in BCC may be interpreted as a molecular indicator of the tumor’s relatively slow growth behaviour.

PANX1, on the other hand, exhibited robust and extensive expression in SCC, particularly in the vicinity of invasive tumour nests and keratin layers. Compared to BCC, SCC is a more aggressive tumour with a greater capacity to metastasize to local lymph nodes and distant organs [44]. In addition to genetic alterations in keratinocytes, changes in stromal cells and the structural support matrix of the dermal stroma in which they are embedded are necessary for the formation and progression of squamous cell carcinomas, especially cutaneous squamous cell carcinoma [45]. PANX1 is involved in purinergic signalling pathways that control cell migration, proliferation, and inflammation, according to studies published in the literature [46], [47], [48]. Consequently, elevated PANX1 expression in SCC may be a marker of enhanced microenvironmental signalling and intercellular communication, both of which are critical for invasive progression. These findings suggest that PANX1 may have a functional role not only in areas of differentiation but also in invasive regions.

From a pathophysiological point of view, it supports the possibility that PANX1 expression might be related not only to epithelial proliferation but also to the invasive and pro-inflammatory behaviour of the tumour [42]. The notable disparity in H-scores seen between SCC and BCC in quantitative QuPath analysis reinforces this possibility. The bioinformatics-based analyses in our study have improved the objectivity and reproducibility of immunohistochemical evaluations. In addition, they minimized observational variation and enabled more precise quantitative determination of expression differences between subtypes. This indicates that quantitative assessments, in addition to morphological characteristics, play a role in distinguishing differences in PANX1 expression.

According to the demographic data in our study, it was determined that differences in PANX1 expression were independent of key patient characteristics such as age and gender. This supports the idea that PANX1 expression is related to tumour biology rather than a variable dependent on patient profile [48]. Given that SCC often develops against a background of chronic sun damage and long-term inflammation[49], the role of PANX1 in inflammatory signaling and intercellular communication [47] appears clinically significant.

We investigated molecular pathways linked to PANX1 expression by performing bioinformatic analyses. These proteins are known to be involved in the processes of inflammation and apoptosis, with CASP3 playing a crucial role in the execution of apoptosis [50] and CASP1 and P2RX7 controlling inflammasome-mediated inflammatory responses [51], 52]. According to Venn diagram analysis, the only overlapping gene between PANX1-interacting proteins and SCC-associated genes obtained from the GeneCards database was CASP3, while no overlapping genes were found for BCC.

It has been shown that during programmed cell death, caspase-3 – an enzyme of vital importance in the apoptosis process – activates purinergic signaling pathways by cleaving PANX1 channels, thereby triggering the release of ATP. It has been proposed that when extracellular ATP activates P2X7 receptors, PANX1 channels open, allowing ATP to be released into the extracellular space and supporting purinergic signaling cascades associated with inflammation [53], 54]. In the tumor microenvironment, ATP release via PANX1 channels acts as a ‘find-me’ signal that promotes the recruitment of immune cells and regulates inflammatory responses [55], [56], [57]. Our bioinformatics analyses supports these findings. Furthermore, as cutaneous squamous cell carcinoma frequently arises from chronic inflammation and increased cell turnover, the relationship between CASP3-mediated apoptosis and PANX1 activation may explain the high levels of PANX1 observed in SCC tissues in our study.

In this regard, the protein-protein docking analysis performed in our work supports the possibility of a direct interaction between PANX1 and CASP3. The docking results revealed that Caspase-3 is positioned close to the DVVD cleavage site of PANX1 and creates a stable binding contact. This indicates that Caspase-3 not only serves an enzymatic function but may also establish a temporary or stable complex with PANX1 to enhance substrate recognition and cleavage. Prior research has demonstrated that caspase-3-mediated cleavage of PANX1 at the C-terminal is essential for channel activation and ATP release [30]. Accordingly, our structural results corroborate the molecular connection between PANX1-mediated purinergic signaling and apoptosis.

The absence of overlapping genes between proteins that interact with PANX1 and BCC-associated genes may indicate fundamental biological differences between these two types of skin cancer. While SCC progression is closely linked to inflammatory signaling and apoptosis mechanisms, BCC carcinogenesis is primarily influenced by dysregulation of the Hedgehog signaling pathway [36]. Therefore, compared to BCC, PANX1-mediated inflammatory and apoptotic signaling pathways may play a more significant role in the pathophysiology of SCC.

ATP-mediated signal transduction is associated with intracellular Ca^2+^ dynamics, and Ca^2+^ signaling is a well-known regulator of caspase-dependent apoptosis [58]. The Venn diagram analysis in our study identified CASP3 as the overlapping gene between PANX1-interacting proteins and SCC-associated genes; this suggests that apoptosis-related processes play a more significant role in SCC compared to BCC. This finding from our study is consistent with other studies showing increased apoptosis activity and inflammatory signaling in SCC [9]. Furthermore, a functional enrichment analysis of the PANX1-associated network identified by STRING revealed notable correlations with ATP-sensitive processes, purinergic signaling components, calcium-related pathways, and caspase-related apoptosis mechanisms. Our study suggests that increased PANX1 expression in SCC may be associated with apoptosis and inflammatory signaling pathways, particularly through interactions with CASP3-related molecular processes.

Consistent with ATP-mediated purinergic signaling associated with interleukin-1β release, the enrichment of interleukin-1-related pathways further supports the link to inflammatory signaling [53]. The involvement of different apoptosis-related pathways varies depending on the tumor type, and comparative analysis suggests that caspase-mediated mechanisms may not always be present. These results imply that PANX1-mediated ATP signaling may regulate inflammation and apoptosis in a context-dependent manner. This evaluation aligns with earlier research on ATP-mediated purinergic signaling linked to the release of interleukin-1β [53].

This study had some limitations. First, the study was retrospectively designed, and clinical outcome data such as recurrence, metastasis, and survival rates were missing. This limited the direct assessment of the prognostic value of PANX1. Second, further confirmatory studies are needed to determine whether PANX1 can be used as a diagnostic marker alone. Third, further functional and molecular studies are needed to determine whether PANX1 directly contributes to the progression of SCC or whether increased PANX1 expression is a secondary manifestation of an invasive phenotype. However, the sufficient number of patients, the fact that the study was conducted in two centers, and the adoption of a methodological approach supported by digital quantitative analysis demonstrate the strengths of our study. Further studies on PANX1 expression in different skin carcinomas and dermatological diseases causing chronic inflammation may show whether this molecule serves as a broader biological or prognostic marker.

## Conclusions

This two-center study, encompassing a very large patient group, revealed significantly higher PANX1 expression in SCC compared to BCC. The findings suggest that PANX1 may be a molecule that reflects subtype-specific biological differences in keratinocyte tumors. Furthermore, the findings indicate a potential role for PANX1 in molecular processes, particularly those associated with the invasive phenotype. Bioinformatic studies have shown that PANX1 interacts with proteins involved in inflammatory and apoptotic signaling pathways, identifying CASP3 as a co-gene associated with SCC. Enrichment was observed in pathways including apoptosis and TNF signaling. In addition, docking results support the close interaction between CASP3 and PANX1, demonstrating the structural basis of caspase-mediated pathways in PANX1 activation. The collective evaluation of the findings indicates that PANX1-related signaling pathways may play a role in the molecular processes underlying the onset and advancement of SCC. Additional investigations, bolstered by clinical outcome data and functional analyses, will enhance the overall comprehension of the prospective diagnostic and prognostic significance of PANX1 in dermatopathology.
